# Gait Training With Motor Relearning Program in Conjunction With Functional Electrical Stimulation in Quadriparesis Secondary to Cervical and Lumbar Myelopathy

**DOI:** 10.7759/cureus.54449

**Published:** 2024-02-19

**Authors:** Aditi Akhuj, Nishigandha P Deodhe, Shrushti Jachak

**Affiliations:** 1 Neurophysiotherapy, Ravi Nair Physiotherapy College, Datta Meghe Institute of Higher Education and Research, Wardha, IND

**Keywords:** cervical spondylotic myelopathy, neurophysiotherapy, neurorehabilitation, quadriparesis, functional electrical stimulation, motor relearning program

## Abstract

Degenerative cervical myelopathy is a frequently encountered age-related pathology following compression of the spinal cord. This case report delineates the clinical manifestation of cervical and lumbar myelopathy in a 78-year-old male patient, characterised by chief complaints of difficulty in moving bilateral upper and lower extremities and difficulty in bed mobility. Motor impairment can manifest in three different presentations, which are paraparesis, hemiparesis, or quadriparesis. The motor relearning program incorporating functional electrical stimulation constitutes a rehabilitative approach used for the restoration of motor function. This study outlines the protocol for the physiotherapy intervention protocol, mainly focusing on gait training. Along with it, balance training, proprioceptive neuromuscular facilitation, etc., were also included. The goal of physiotherapy rehabilitation was to improve the patient's ability to do tasks related to daily living. The outcome measures used were the dynamic gait index, functional independence measure, and Modified Japanese Orthopaedic Association score. We document significant increases in muscular tone and power, improved active range of motion, enhancements in gait parameters, and notable advancements in the individual's functional independence through the implementation of a physiotherapeutic regimen.

## Introduction

The term "myelopathy" refers to any pathology that results in a spinal cord-related neurologic impairment [1,2]. Degenerative cervical myelopathy (DCM) is known as a spinal cord disorder characterised by chronic spinal cord compression brought on by degenerative alterations to the vertebral column [3]. Clinical manifestations associated with cervical myelopathy, such as diminished hand dexterity, postural instability, compromised gait function, sensory deficits, and disturbances in bladder control, align with the specific level of injury within the cervical spine. Functional impairment is a major issue for DCM patients. Around 35-45% of individuals affected by DCM experience limitations in ambulation, with 35-39% continuing to exhibit gait disturbances even following medical or surgical interventions [4]. The L4-L5 and L5-S1 levels of the lower spine are the sites of the most frequent disc herniations and spondylotic abnormalities. Merely 5% of herniations in the lumbar disc manifest at a more superior location, predominantly at the L3-L4 vertebral level [5]. In general, motor deficiencies caused by neurological illnesses are common, significantly impact an individual's personal and professional life, and exert a substantial impact on the healthcare and socioeconomic systems [6,7].

Therapeutic interventions encompass physical therapy, cervical orthosis application, conservative observation, and surgical decompression with or without adjunct fusion. Evidence-derived guidelines and scientific literature advocate surgical decompression as the recommended intervention for cases of moderate to severe DCM. Surgical decompression is regarded as the most effective course of action. Surgical treatments, including anterior or posterior decompression combined with arthrodesis, arthroplasty, or laminoplasty, should be taken into consideration for individuals whose cervical myelopathy is persistently progressing [8]. Surgical decompression has the potential to prevent the progression of myelopathy and enhance neurological function, quality of life, and overall results. About 70% of patients get favourable results classified as excellent or good after surgical decompression with either an anterior or posterior technique [9].

The motor relearning program (MRP), as conceptualised by Carr and Shepherd, posits that the process of motor relearning necessitates repeated, task-specific training. The implementation of repetitive exercises with an emphasis on task-oriented activities within the framework of MRP, coupled with the adjunctive application of functional electrical stimulation (FES), may be employed to enhance functional movements [10]. MRP is divided into seven categories that correspond to the main functions of daily living. These sections are primarily grouped together as upper limb function, orofacial function, motor tasks done while standing and sitting, walking, and standing up and sitting down [11]. Electrical stimulation is the process of depolarizing a neuron or muscle (if a muscle has been denervated) with an external electrical current in order to create an action potential, which, when propagated, causes a contraction of the muscle. FES is a technique that uses neuromuscular electrical stimulation in a preset order to cause contractions in weak muscles so that the person may perform functional tasks including walking, stepping, standing, and doing routine activities. FES involves a specific electrical stimulation sequence that is either pre-programed into the device or integrated into the device through a triggering mechanism [12]. The anticipated outcome is that, following training with the FES, patients will exhibit the capacity to independently execute the specific activities for which they were trained without relying on the FES; that is, patients are expected to regain voluntary function [13].

The principal aim of neurorehabilitation training is to facilitate gait rehabilitation or recovery, as neurological disorders often result in individuals exhibiting gait abnormalities. These abnormalities encompass muscle weakness, uneven step length, diminished walking speed, an uncoordinated inter-limb walking pattern, compromised balance, and reduced force generation [14]. In addition to assisting the injured in achieving maximal function, rehabilitation can help patients reclaim their sense of well-being and a fulfilling amount of independence. Rehabilitation not only maximises healing but also reduces the risk of subsequent medical disorders, including sublesional osteoporosis, neurogenic bowel and bladder dysfunction, spasticity, neuropathic pain, and cardiovascular disease. Additionally, it is well recognised that during the crucial phase of recovery, rehabilitative exercises might enhance neuroplasticity [3]. This manuscript presents a case report delineating the clinical details of a 78-year-old male with quadriparesis secondary to cervical and lumbar myelopathy, and the goal of this paper was to prove the importance of physiotherapy in this condition.

## Case presentation

Patient information

A 78-year-old male patient visited the hospital with complaints of weakness in the right upper limb, followed by the left upper limb, and after 10 days in the right lower limb, followed by the left lower limb for six months. The onset of symptoms was insidious, with a gradual and progressive nature. He also complained of headaches for eight days. There was no recorded history of falls, back trauma, or recent heavy weight lifting. Following an assessment and evaluation, the patient underwent investigations such as magnetic resonance imaging (MRI) of the cervical and lumbar spine, which was suggestive of disc protrusions in the cervical and lumbar spine with disc bulges at C5-C7 disc levels and L2-3 to L5-S1. The patient was referred to physiotherapy, and physiotherapy rehabilitation was initiated using a tailor-made protocol designed to address the specific requirements of the patient.

Clinical findings

The patient's written and verbal consent was acquired before conducting the physical examination. He demonstrated consciousness, cooperation, and orientation to person, place, and time. The patient maintained hemodynamic stability and was afebrile. The patient was observed in a supine-lying position and displayed lethargy. Muscle strength was 3-/5 according to Kendall's grading in both upper and lower limbs. During a neurological assessment, both superficial and deep sensations were found to be intact. However, there was a reduction in tone. Notably, all deep tendon reflexes demonstrated diminished activity, and no bony tenderness was present. The patient exhibited difficulty standing and walking. As per the functional independence measure (FIM) assessment, the individual necessitated moderate assistance for fundamental activities of daily living (ADLs), including tasks such as eating, bathing, toileting, and transferring, in addition to instrumental ADLs such as handling and transportation.

Diagnostic assessment

**Figure 1:**
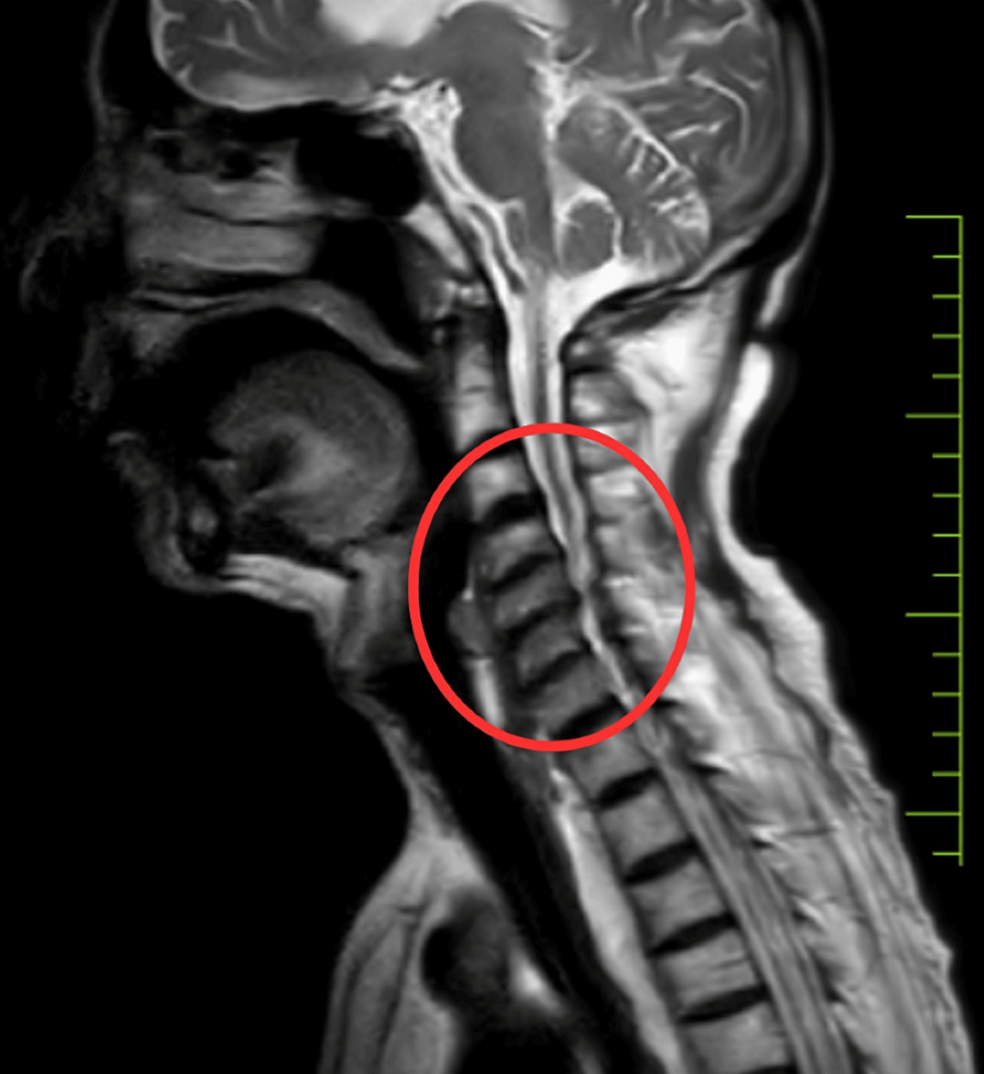
MRI of the cervical spine The circle shows C4-C6 parenchymal disc bulge indenting over the anterior thecal sac causing narrowing of neural foramen, and C6-C7 level posterior diffuse bulge indenting over the anterior thecal sac MRI: magnetic resonance imaging

The patient underwent a clinical and radiological examination. An MRI of the cervical spine showed the features of cervical spondylosis: a C4-C6 parenchymal disc bulge indenting over the anterior thecal sac, causing narrowing of the neural foramen, and a C6-C7-level posterior diffuse bulge indenting over the anterior thecal sac (Figure 1).

**Figure 2:**
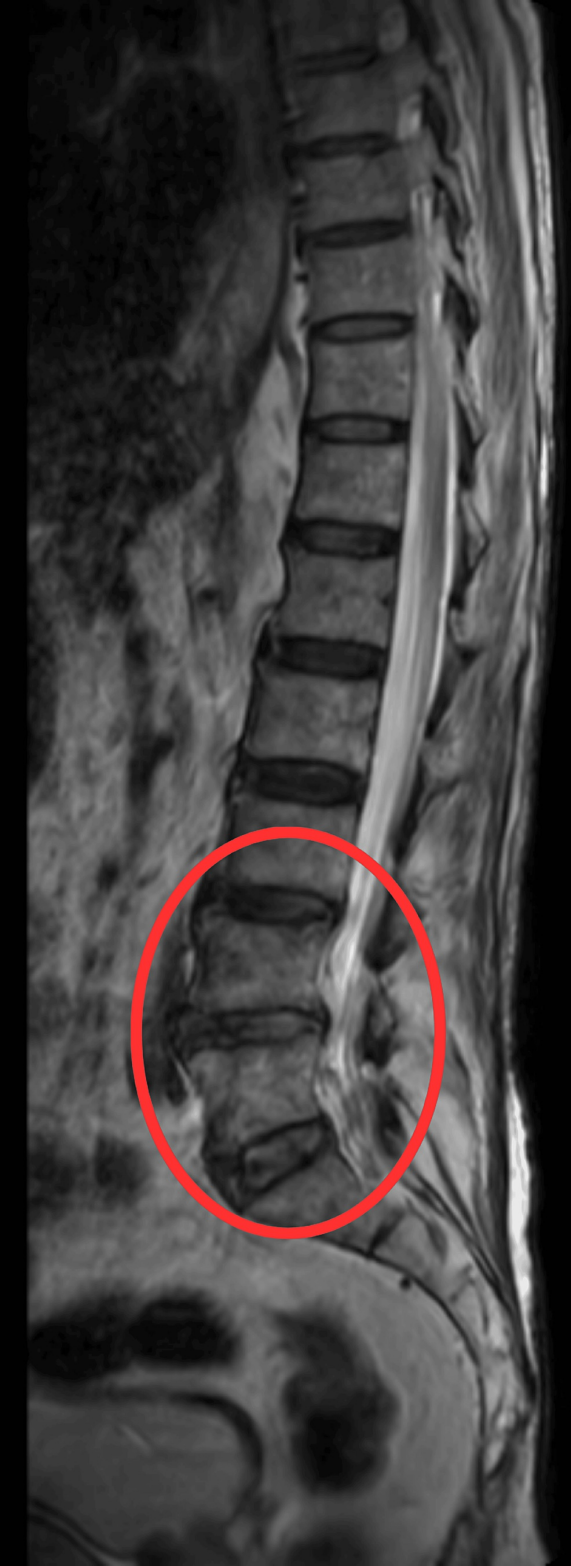
MRI of the whole spine The circle shows L2-3 to L5-S1 level mild posterior disc bulge seen indenting anterior thecal sac, and no central canal stenosis MRI: magnetic resonance imaging

MRI screening of the whole spine was done, which revealed an L2-3 to L5-S1-level mild posterior disc bulge seen indenting the anterior thecal sac and no central canal stenosis (Figure 2).

Physiotherapy intervention

The patient underwent a physiotherapy intervention for three weeks. First, the patient and his family were educated to build up and keep up the patient’s positive attitude regarding treatment to facilitate an early recovery. The importance of physical therapy intervention was discussed with the patient and his family, and they received a thorough explanation of the patient's condition. The patient was given diaphragmatic breathing exercises to improve the ventilation and oxygenation of the lung. Facilitation techniques such as proprioceptive neuromuscular facilitation (PNF) rhythmic initiation (D1 and D2 patterns) were initiated for bilateral upper and lower limbs (10 repetitions x 1 set) to enhance muscular tone, progressing to PNF dynamic reversals (slow reversals, stabilising reversals, and rhythmic stabilization). To enhance the strength of muscles, PNF hold-relax for the upper and lower limbs (10 reps x 1 set) along with strengthening exercises with a ½ kg weight cuff were given. Core stability exercises included pelvic bridging exercises (10 repetitions with a five-second hold, performed for a single set), half push-ups in prone lying (six repetitions in one set), and progressing to full push-ups in prone lying (10 repetitions in one set). Arm movements were trained, followed by MRP for improving hand function through task-oriented activities such as opening and closing bottle lids, picking and drinking water from glass, putting puzzle pieces in order, reaching and repositioning water glass, moving small objects between containers, turning handgrips on doors, reading magazines, and turning pages of newspapers or books.

Balance and gait retraining were given to gain static and dynamic balance by MRP. The static and dynamic balance were improved by giving perturbations, reach-outs, weight shifting, sit-to-stand, and stepping strategies. The exercises were administered in three positions: supine hip, knee flexed, and ankle dorsiflexion. Ankle dorsiflexion was carried out at different angles of hip-knee flexion, in sitting ankle dorsiflexion with hip-knee flexion, and at different degrees of knee extension.

The patient received MRP with FES for lower limbs for a duration of 15 minutes along with MRP spanning one hour, administered on a frequency of five days per week over a three-week period. FES was administered to the dorsiflexors and plantarflexors of the lower limb. FES was given with a 40 Hz current for 25 minutes, alternating between eight seconds of contraction and relaxation. The focus was on the voluntary attempt by the patient to execute the movement (gait) while receiving stimulation from the FES.

Analysis of the Walking-Stance Phase

Lack of extension of the hip and dorsiflexion of the ankles, lack of controlled knee flexion-extension, lateral horizontal shift of the pelvis. Lack of knee flexion at toe-off, lack of hip flexion, lack of knee extension, plus ankle dorsiflexion on heel strike in the swing phase. The patient lacked an idea of the sequencing of components and the timing of walking. Inability to bear weight through his leg in normal alignment with hip extension, controlled knee extension, and stepping forward. Difficulty in shifting the centre of gravity laterally. Inability to extend the affected hip, lack of knee control throughout the stance phase, lack of knee flexion at the toe-off, lack of active dorsiflexion at the end of the stance phase, inability to clear the ground and dragging of the foot, wide base of support while walking due to poor balance.

Practice of Missing Components

The main focus was on training hip extension, knee control throughout the stance phase, lateral horizontal pelvic shift, flexion of the knee at the start of the swing phase, and knee extension and foot dorsiflexion at the heel strike. The patient was taken into the appropriate position; instructions were given to maintain proper body alignment, and with the demonstration of verbal feedback, he was taught to activate the muscles needed at each phase of gait.

Practice of Walking and Transference of Training into Daily Life

Normal standing with feet a few inches apart, one step forward with and then backwards, forward bending and dorsiflexion, standing against the wall, feet a few inches away from it, backwards walking, stepping over objects of different heights combined with other activities, varying the speed of walking, climbing up and down stairs. Initially, the patient was trained with walking aids such as parallel bars and a cane.

Follow-up and outcomes measures

After three weeks of therapeutic treatment, a follow-up was carried out which showed improved muscle strength (Table 1).

**Table 1:** Pre- and post-intervention muscle strength of upper limb and lower limb 3-: some but not complete ROM against gravity; 4: full ROM against gravity, moderate resistance; 5: full ROM against gravity, maximal resistance ROM: range of motion

Joint	Muscles	Pre-intervention		Post-intervention	
		Right	Left	Right	Left
Upper extremity					
Shoulder	Flexors	3-/5	3-/5	4/5	4/5
	Extensors	3-/5	3-/5	4/5	4/5
	Abductors	3-/5	3-/5	4/5	4/5
Elbow	Flexors	3-/5	3-/5	4/5	4/5
	Extensors	3-/5	3-/5	4/5	4/5
Lower extremity					
Hip	Flexors	3-/5	3-/5	4/5	4/5
	Extensors	3-/5	3-/5	4/5	4/5
	Abductors	3-/5	3-/5	4/5	4/5
	Adductors	3-/5	3-/5	4/5	4/5
Knee	Flexors	3-/5	3-/5	4/5	4/5
	Extensors	3-/5	3-/5	4/5	4/5
Ankle	Dorsiflexors	3-/5	3-/5	4/5	4/5
	Planatrflexors	3-/5	3-/5	4/5	4/5

Table 2 depicts the pre and post-intervention muscle tone according to the tone grading scale.

**Table 2:** Pre- and post-intervention tone (TGS) 1+: diminished; 2+: normal TGS: tone grading scale

Joint	Muscles	Pre-intervention		Post-intervention	
		Right	Left	Right	Left
Upper extremity					
Shoulder	Flexors	1+	1+	2+	2+
	Extensors	1+	1+	2+	2+
	Abductors	1+	1+	2+	2+
Elbow	Flexors	1+	1+	2+	2+
	Extensors	1+	1+	2+	2+
Lower extremity					
Hip	Flexors	1+	1+	2+	2+
	Extensors	1+	1+	2+	2+
	Abductors	1+	1+	2+	2+
	Adductors	1+	1+	2+	2+
Knee	Flexors	1+	1+	2+	2+
	Extensors	1+	1+	2+	2+
Ankle	Dorsiflexors	1+	1+	2+	2+
	Planatrflexors	1+	1+	2+	2+

All the deep tendon reflexes were normal post-intervention, as detailed in Table 3.

**Table 3:** Pre and post-intervention deep tendon reflexes +: diminished reflex; ++: normal reflex; +++: exaggerated reflex

Reflexes	Pre-intervention		Post-intervention	
	Right	Left	Right	Left
Bicep jerk	+	+	++	++
Triceps jerk	+	+	++	++
Knee jerk	+	+	++	++
Ankle jerk	+	+	++	++

Outcome measures, including FIM, dynamic gait index (DGI), Modified Japanese Orthopaedic Association (mJOA) score, and gait parameters, were evaluated pre- and post-treatment (Table 4).

**Table 4:** Outcome measures mJOA: Modified Japanese Orthopaedic Association; DGI: dynamic gait index; FIM: functional independence measure

Outcome measures	Pre-intervention	Post-intervention
mJOA score	6/18	16/18
DGI	2/24	20/24
Gait parameters	Step length: 30 cm, stride length: 60 cm, cadence: 50 steps/min	Step length: 55 cm, stride length: 100 cm, cadence: 80 steps/min
FIM	14/126	70/126

## Discussion

The term "myelopathy" refers to any condition that results in a spinal cord-related neurologic impairment. Among the most typical reasons for myelopathy, one is degenerative spine disease. This may arise due to the compression of the spinal cord in the cervical, lumbar, or, less commonly, thoracic spine, potentially caused by an osteophyte or extruded disc material. Trauma to the cervical and lumbar spines can cause quadriparesis, a difficult and debilitating disorder requiring extensive rehabilitation approaches. Evidence-derived guidelines and scientific literature advocate surgical decompression as the recommended intervention for cases of moderate to severe DCM. Surgical decompression is regarded as the most effective course of action. Physiotherapy encompasses therapeutic interventions aimed at enhancing limb functionality, promoting trunk stabilisation, and facilitating gait training through the implementation of targeted exercise regimens. Research has demonstrated that several of the therapeutic goals in DCM, including walking, balance, and pain, are also common to spinal cord injury and, to a greater extent, stroke [3]. The principal aim of neurorehabilitation training is to facilitate gait rehabilitation or recovery, as neurological disorders often result in individuals exhibiting gait abnormalities. These abnormalities encompass muscle weakness, uneven step length, diminished walking speed, an uncoordinated inter-limb walking pattern, compromised balance, and reduced force generation.

The patient showed improved strength of muscles and speed of movement after receiving treatment with the PNF technique. Cayco et al. found that a PNF strategy in conjunction with neuroplasticity principles was a dependable and successful means of helping older patients with chronic stroke regain their motor outputs. Certain PNF techniques, in particular, can enhance strength, dexterity, and movement speed in non-spastic muscle areas [15]. Physical therapy, according to Shroff et al., aids people with spinal cord injury with day-to-day disability management. Along with mobilisation exercises, it entails activating the nerves and muscles below the injured site [16].

Motor relearning is conceptualised as a series of processes linked to repetitive practice or experiential engagement, resulting in enduring alterations in the proficiency of movement capabilities. Research conducted with stroke populations has demonstrated that the implementation of MRP, task-specific training, and task-related training incorporating specific exercises to strengthen the paretic muscles contributes to enhancements in locomotion. This includes the ability to bear lower limb weight while sitting, achieving an upright standing posture, and improving gait parameters, ultimately leading to an improvement in the quality of life [17]. According to one study, FES therapy has been shown to improve the paretic hand's motor function. Additionally, functional exercise combined with tailored electrical therapy for the paretic upper limb may improve neuroplasticity in chronic stroke patients, which is shown as corticospinal facilitation, and also lead to moderate improvements in the affected limb's voluntary motor control [18]. Ullah et al. studied the effect of MRP along with FES and found that it substantially improves the function of the upper extremities in people with subacute strokes [10]. Cheng et al. studied the effect of perturbation-based balanced training (PBT) in cervical myelopathy patients and discovered it to be beneficial for lower limb function and balance [19]. Likewise, we used PBT, and it had a favourable impact on lower limb functioning and balance. Sheffler et al. evaluated the effectiveness of FES on motor relearning in lower limb hemiparesis and concluded that in chronic stroke survivors missing dorsiflexion, it improved functional mobility [20]. Similarly, by applying FES to our patient, we found improved motor function and functional mobility.

## Conclusions

With the global rise in myelopathy cases, conducting thorough and effective studies on diverse treatment modalities is crucial. Surgical decompression with or without concomitant fusion, physical therapy, and conservative surveillance are all considered therapeutic approaches. This case study underscores the significance of a well-structured three-week physiotherapeutic intervention complemented by the MRP with FES, showcasing enhanced treatment outcomes. It emphasises the remarkable benefits of this approach in facilitating motor relearning, improving muscle tone and strength, addressing balance and gait impairments, enhancing overall quality of life, and promoting functional independence for patients dealing with cervical and lumbar myelopathy.
